# The Antioxidant, Antimicrobial, and Antitumor Proprieties of Flavonol-Rich Extracts from *Allium ursinum* (Wild Garlic) Leaves: A Comparison of Conventional Maceration and Ultrasound-Assisted Extraction Techniques

**DOI:** 10.3390/ijms252312799

**Published:** 2024-11-28

**Authors:** Kinga Oravetz, Zorita Diaconeasa, Rahela Carpa, Elena Rakosy-Tican, Daniel Cruceriu

**Affiliations:** 1 Centre for Systems Biology, Biodiversity and Bioresources, Department of Molecular Biology and Biotechnology, Faculty of Biology and Geology, Babes-Bolyai University, 5-7 Clinicilor Street, 400006 Cluj-Napoca, Romania; kinga.oravetz@ubbcluj.ro (K.O.); elena.rakosy@ubbcluj.ro (E.R.-T.); daniel.cruceriu@ubbcluj.ro (D.C.); 2 Department of Chemistry, Biochemistry and Molecular Biology, University of Agricultural Sciences and Veterinary Medicine, 3-5 Calea Mănăştur, 400372 Cluj-Napoca, Romania; zorita.sconta@usamvcluj.ro; 3 Institute for Research-Development-Innovation in Applied Natural Sciences, Babes-Bolyai University, 30 Fântânele St., 400294 Cluj-Napoca, Romania; 4 The Oncology Institute “Prof. Dr. Ion Chiricuta”, Department of Genetics, Genomics and Experimental Pathology, 34-36 Republicii Street, 400015 Cluj-Napoca, Romania

**Keywords:** wild garlic, polyphenols, alliin, antibacterial activity, cytotoxicity

## Abstract

Despite the growing interest in using natural compounds for disease prevention and treatment, *Allium ursinum* (wild garlic), known for its therapeutic properties, has not been extensively studied for its chemical composition and biological activities. Therefore, this study aims to explore the in vitro antioxidant, antibacterial, and antitumor activities of *A. ursinum* extracts according to their functional phytochemical profile, while assessing whether ultrasound-assisted extraction (UAE) enhances bioactive properties in comparison to conventional maceration (CM). Both extracts were characterized by spectrophotometric methods and LC-ESI+-MS. The antioxidant activity was assessed via the CUPRAC and hydrogen peroxide scavenging assays, the antimicrobial properties via the disk-diffusion method against five pathogenic strains, and the antitumor activity via the MTT assay on four cancer cell lines. The major constituents of the methanolic extracts from leaves were kaempferol derivatives and alliin. The quercetin derivative rutin was also found. Maceration assisted using UAE yielded 20% more bioactive compounds in comparison to CM alone. Employing UAE in the extraction significantly increased antioxidant and antimicrobial proprieties, in line with its chemical composition. The antitumor cytotoxic activity was low to moderate, regardless of method, as explained by the absence of highly cytotoxic compounds. Wild garlic extracts possessed strong antioxidant and substantial antibacterial activities.

## 1. Introduction

In the recent past, the use of natural compounds in the prevention and treatment of several diseases including cancer and bacterial infectious diseases has gained much attention [1]. Some of the oldest plants used intensively since antiquity for their therapeutic properties are plants belonging to the Alliaceae family. Garlic, onions, leeks, and chives, all belonging to this family, are well known for their antioxidant, antibacterial, anticancer, hepatoprotective, or cardioprotective activities [2], with most of these biological effects being related to their rich content in organosulfur compounds [3].

Despite the numerous demonstrated health benefits of this family, some of the wild members of *Allium* genus have not yet been intensively studied for their chemical composition and biological activities [4]. One such understudied plant is *Allium ursinum*, also known as wild garlic, bear garlic, or elephant garlic. Wild garlic is native to Asia and Europe, especially Eastern Europe, including Romania, being an important diet component during springtime [5,6]. Unlike other species belonging to this genus, wild garlic has not yet been cultivated as it has demanding requirements, especially environmental conditions which are very hard to reproduce [7]. *Allium ursinum* is also difficult to maintain using micropropagation because of its specific soil requirements, slow growth, and poor germination rate [8]. Thus, this species had not garnered much interest until recently, and information on its phytochemistry and pharmacological activity is scarce.

Based on the limited data available, *A. ursinum*, much like other *Allium* species, owes its biological activities to polyphenols and sulfur-containing compounds (OSCs). Interestingly, it was previously demonstrated that wild garlic has about seven times more active sulfur compounds than cultivated garlic [9]. Therefore, different extracts of this species were proved to possess cardioprotective, antihypertensive, anticarcinogenic, antimicrobial, and antioxidant activities [10,11,12,13,14], retaining most of the health-related benefits of garlic. However, it has been suggested that the biological activity of *A. ursinum* is weaker, necessitating higher doses for effectiveness [15]. In addition to OSCs and polyphenols, wild garlic contains volatile oils, steroidal glycosides, and dietary fibers, which also contribute to its pharmacological activity [4,16,17]. Considering all of this, it is not surprising that wild garlic is gaining significant interest for its potential in preventing and treating various diseases [11].

However, data from the literature state that the date of harvest, soil, and geographical location significantly influence the chemical composition of *Allium ursinum* [17,18]. Moreover, the quality and quantity of bioactive content may be affected by the extraction technique and the solvent used, thereby affecting the overall biological effects of the obtained extract [18]. Maceration is a traditional extraction method valued for its simplicity and effectiveness in producing high-quality extracts, making it ideal for analyzing a wide range of phytochemicals [19]. In contrast, ultrasound-assisted extraction (UAE) is a modern technique that enhances phytochemical extraction by disrupting plant cell walls and bypassing limitations related to component polarity or molecular weight [20]. However, some studies suggest that high ultrasonic power or frequency can produce free radicals, leading to the degradation of polyphenols [21]. Conversely, other research indicates that UAE can enhance phenolic content and antioxidant activity, improving bioavailability [22]. Therefore, the aim of this study was to examine the in vitro antioxidant, antibacterial, and antitumor activities of methanolic extracts obtained from the leaves of *A. ursinum* from the spontaneous flora in Romania, while exploring if ultrasound-assisted extraction in combination with maceration (UAE+CM) enhances its bioactive properties compared to conventional maceration alone (CM), in line with the phytochemical profile of its formulations.

## 2. Results and Discussion

### 2.1. The Extraction Yields of CM and UAE Methods for A. ursinum Leaves

The extracts of *A. ursinum* leaves were obtained in methanol 70%, starting with 4 g of dried plant material, using two alternative extraction techniques: conventional maceration (CM) and ultrasound-assisted extraction followed by maceration (UAE+CM). Methanol 70% was selected as the solvent, due to its capability of extracting a wide variety of polar phytoconstituents, with higher efficiency in comparison to other solvents [23]. The extraction yield for the CM method was 41.32%, whereas the UAE+CM extract had a yield of 45.82%. Consistent with previous studies [24], our findings show that inclusion of UAE in the extraction can lead to higher quantities of bioactive compounds compared to traditional methods.

### 2.2. Phytochemical Characterization of A. ursinum Extracts

Total phenolic (TPC) and flavonoid (TFC) contents of the extracts were determined spectrophotometrically. The CM was characterized by a TPC of 47.63 mg GAE (gallic acid equivalent)/100 mL and a TFC of 4.68 mg QE (quercetin equivalent)/100 mL. The presence of phenolic compounds, including flavonoids, in *A. ursinum* leaves was previously reported [25,26]. Moreover, according to Kovarovic et al. (2019), *A. ursinum* leaves contain statistically significant higher content of total polyphenols compared to flowers and bulbs [27]. The UAE+CM extract had similar TPC and TFC, with contents of 48.14 mg GAE/100 mL and 4.59 mg QE/100 mL, respectively. Although, Gîtin et al. 2012 found that by using UAE, the level of flavonoid was much higher than the level of flavonoid obtained via CM; in our study, the differences between the two extraction methods are minor and might indicate similar efficiency in extracting polyphenols [6]. However, it is important to consider that these quantification methods are still quite rudimentary.

**Figure 1:**
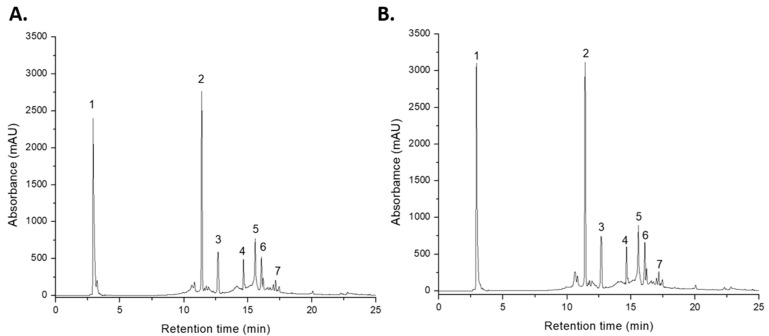
LC chromatograms showing the peaks obtained for both extracts from *Alium ursinum* leaves, recorded at 340 nm, containing all seven identified compounds. (**A**) Extract obtained through conventional maceration (CM) and (**B**) extract obtained through ultrasound-assisted extraction (UAE).

Phenolic and sulfoxide compounds in both *Allium ursinum* extracts were tentatively identified using LC-UV-ESI+-MS and further semi-quantified using standard curves (gallic acid—thiosulphinates and rutin—flavonols). Phenolic and sulfoxide compounds in both *Allium ursinum* extracts were identified using LC-ESI+-MS and quantified using standard calibration curves. Specifically, alliin content was determined using a gallic acid calibration curve (R^2^ = 0.9978) over a concentration range of 10–100 μg/mL for thiosulfinates, while flavonol content was assessed using a rutin calibration curve (R^2^ = 0.9981) with the same concentration range. Of the seven major peaks (Figure 1), six peaks belonged to flavonols, while one peak corresponded to the sulfoxide alliin (peak 1). The tentative identification of phenols and sulfoxides in the methanolic extracts of *A. ursinum* leaves, based on retention time, wavelengths of maximum absorption, and mass spectra, is presented in Table 1.

Alliin is found in high concentration in *Allium* plants, and it is the precursor of allicin which is formed via the action of alliinase enzyme upon crushing [28]. Allicin is known for its antimicrobial and anticancer properties; thus, the presence of alliin adds substantial therpeutic value to *A. ursinum* [29]. The time of harvest is known to affect alliin content, with the highest amount being found in extracts from leaves that were harvested between March and April [28]. Processing treatment also affects alliin content, and it was reported that dried wild garlic leaves contain 0.7–1.7% alliin, whereas fresh wild garlic leaves contain smaller amounts (0.25–1.15%) [30]. In accordance with these data, in this study, substantial amounts of alliin were observed in both extracts obtained from air-dried *A. ursinum* leaves that were harvested in April.

The six flavonols found in the extracts were tentatively identified as kaempferol-glucosyl-glucosyl-rhamnoside (peak 2), kaempferol-diglucoside (peak 3), kaempferol-galactosyl-rhamnoside (peak 4), kaempferol-rutinoside (peak 5), quercetin-rutinoside (peak 6), and kaempferol-glucoside (peak 7). Our results are concordant with other recent studies, where most of the major phenolic compounds detected in extracts of *A. ursinum* were kaempferol glycosides [7,31,32]. Stupar et al. (2022) also found notable concentrations of kaempferol derivatives ranging from 1.97 to 89.19 μg/mL in extracts obtained via subcritical water extraction [14]. The quercetin derivative, rutin, was found in significant quantities in both extracts and was also previously reported as part of the biochemical profile of *A. ursinum* [33]. Research indicates that parts of the plant used for extraction significantly influence flavonol concentrations. For instance, Oszmiański et al. identified 21 phenolic compounds in different organs of *Allium ursinum*. The kaempferol derivatives were found to be predominant in the leaves compared to the seeds and stalks [32].

Alliin alongside flavonols like quercetin and kaempferol which also have potent antioxidant and antimicrobial effects may produce synergistic effects that enhance the overall therapeutic potential of this plant [34,35].

The total amount of phenolic compounds in CM extract was 12.539 mg/mL (Table 1). Among them, the major components were kaempferol-glucosyl-glucosyl-rhamnoside (29.49% of all compounds) and kaempferol-rutinoside (17.75% of all compounds). Besides them, alliin represented 26.05% of all compounds found in the extract. Similar results were obtained for the UAE+CM extract (Table 1) in terms of the diversity and proportion of each constituent. Although both CM and UAE+CM extracts contained substantial amounts of the same flavonols and alliin, the UAE addition to the extraction method resulted in approximately 20% higher concentrations in both categories of the compounds, demonstrating again that the UAE technique extracts higher quantities of bioactive compounds compared to traditional maceration.

### 2.3. The Antioxidant Activity of A. ursinum Extracts

The determination of antioxidant capacity of *A. ursinum* extracts appears to be of great importance, and it has been the subject of various studies, revealing its potential health benefits and therapeutic applications [36]. Several studies confirmed that there is a correlation between phenolic and organosulphur compounds and antioxidant activity. For example, according to Pavlović et al., *A. ursinum* extracts possess superior antioxidant capacities compared to other wild plants belonging to the genus *Allium*, suggesting that its specific phytochemical profile contributes significantly to its antioxidant capacity [15]. Similarly, Rankovic et al. noted that the methanolic extract of *A. ursinum* has significantly high levels of phenolic acids and flavonoids, positively correlating with its antioxidant activity [37].

Moreover, data from the literature suggest that the antioxidant capacity is influenced by the maturity stage of the plant at the time of harvesting. Toth et al. (2018) found that TPC and antioxidant activity in the leaves of wild garlic increases with maturity [38]. Beside the stage of maturity, the extraction method applied can substantially affect the antioxidant activity. This observation was made by Tomšik et al. 2016 who optimized the extraction conditions in order to obtain maximum yields for each of the following observed response: TPC, TFC, and antioxidant capacity. To obtain a higher antioxidant activity, increasing the temperature and lowering the ultrasonic power are essential [39].

In our study, the antioxidant activity was tested using two methods: the CUPRAC and H_2_O_2_ scavenging activity. Due to their differing underlying principles, the two methods complement each other and give a more holistic view of the sample’s antioxidant potential across different oxidative stressors. When the antioxidant activity was measured using the CUPRAC method, the UAE extract possessed higher reducing activity (87.07 µM Trolox/mL), but not significantly different from the value obtained via CM (85.68 µM Trolox/mL). On the other hand, when tested using the H_2_O_2_ scavenging assay, the extract obtained via UAE had a significantly stronger antioxidant activity (37.71 µmol Trolox/L) in comparison to the CM extract (22.82 µmol Trolox/L). As far as we know, this is the first time that antioxidant capacity of wild garlic extracts was measured using H_2_O_2_ scavenging activity. These data align with our previous results, which show that the UAE extract is richer in polyphenols (Table 1), compounds known for their strong antioxidant activities [40]. While it is difficult to draw an accurate parallel between the antioxidant capacity of different *A. ursinum* extracts because of the variations in assays and the units of measurement used to measure and express the total antioxidant capacity of a sample, a study by Petkova et al. (2021) reported similar findings for *A. ursinum* extracts obtained from flowers when using the CUPRAC assay [41].

### 2.4. The Antimicrobial Activity of A. ursinum Extracts

The antimicrobial activity of *A. ursinum* extracts against *Staphylococcus aureus*, *Enterococcus faecalis*, *Streptococcus mutans*, *Porphyromonas gingivalis*, and *Escherichia coli* was evaluated using the disk-diffusion method. UAE and CM extracts in both methanol 70% and DMSO as vehicles were tested in comparison to the corresponding negative controls. The *A. ursinum* derived extracts demonstrated antibacterial activity against all tested bacterial strains in comparison with the vehicle solvents (methanol 70% and DMSO), which showed zero growth inhibition (Table 2). To our knowledge, this is the first time *A. ursinum* extracts were assessed on *S. mutans* and *P. gingivalis*.

**Figure 2:**
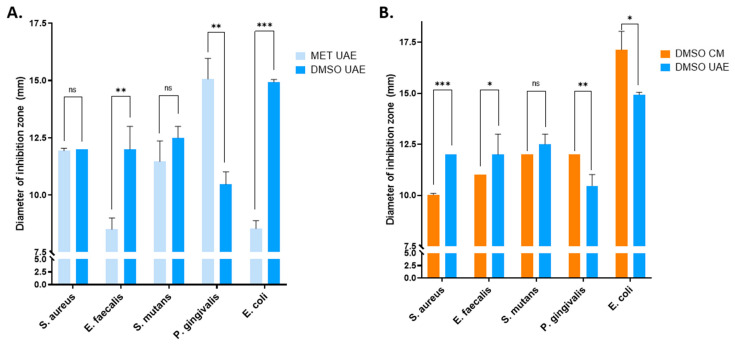
Antimicrobial activity of *Allium ursinum* leaves extracts obtained through conventional maceration (CM) and ultrasound-assisted extraction (UAE) on Gram+ and Gram− pathogenic microorganisms. (**A**) Comparison between the UAE extracts in methanol 70% vs. DMSO solvents, in terms of the diameter of inhibition zone (DIZ), for all bacterial strains. (**B**) Comparison between the UAE extract and the CM extract in DMSO solvent, in terms of the diameter of inhibition zone (DIZ), for all bacterial strains (* 0.05 > *p* ≥ 0.01 significant; ** 0.01 > *p* ≥0.001 very significant; *** *p* < 0.001 highly significant; ns, no significant).

The strongest activity was registered against Gram-negative bacteria, mainly *E. coli* and *P. gingivalis*, with DIZ as high as 17.1 mm and 15.1 mm, respectively. In these cases, the antimicrobial activity of the extracts is similar to that of gentamicin, which served as the positive control. This seems to contradict with previous reports, which suggest that wild garlic extracts are more effective against Gram-positive than Gram-negative bacteria, with strains of *E. coli* being less sensitive than *E. faecalis* [14] or *S. aureus* [12,42,43]. However, none of these studies used the UAE technique to produce the extracts, nor used DMSO as the final solvent; both of these parameters strongly influence the results (Table 2, Figure 2).

When methanol 70% was used as a solvent in a conventional maceration extraction procedure, our results indeed show that the *A. ursinum* extract has the lowest antimicrobial activity against *E. coli* (DIZ = 8.0) in comparison to all the Gram-positive bacterial strains, as expected. On the other hand, using DMSO as a vehicle, which is known to be a very good solvent for bio-organic compounds in high concentrations [44], or employing the UAE method, which was shown in this article to extract higher quantities of bioactive compounds, strongly increased the antimicrobial activity of the extract against *E. coli* (Table 2).

Similar patterns were observed for all Gram-positive bacterial strains used in this study; using DMSO as a vehicle or UAE as the extraction method significantly increased the observed antimicrobial activity of the extracts (Figure 2). Consequently, for all bacterial strains, the DMSO UAE extract had the strongest antimicrobial activity.

The biochemical composition of the extracts explains the registered antimicrobial activity of the extracts, as kaempferol and quercetin derivates [45], as well as alliin [46], are known for their antibacterial proprieties. Different thiosulfinates and polyphenolic compounds are known to generate the antimicrobial activity of the species in the *Allium* genus [14,47]. It was observed that there are multiple sites, at the cell scale, involved in the complex antibacterial process displayed. Gallic acid exhibits strong antibacterial capabilities via the irremediable disturbance of the membrane of *S. aureus* and *E. coli* [48]. This antimicrobial activity is based on the location of the double bonds in the ring and the position of carboxyl and hydroxyl functional groups. Some studies also validated the antimicrobial action of kaempferol and catechin [49,50]. These two derivates and alliin are present in our *A. ursinum* extract, confirming its antimicrobial activity. The potential of *A. ursinum* as a natural antibacterial agent is supported by a large number of research. Regarding the antibacterial activity of *A. ursinum* extracts against different Gram-positive and Gram-negative bacteria, prior research has produced contradictory findings [14,18,43,51]. As previously said, this can be explained by the extraction method, plant origin, plant component, and the isolation of various active chemicals utilizing various solvents during extraction. Thus, acetone, chloroform, ethyl acetate, n-butanol, and water extracts of fresh *A. ursinum* flowers and leaves were examined for their antibacterial activity against a variety of Gram-positive and Gram-negative bacteria by Ivanova et al. [43]. While acetone and chloroform extracts containing organosulfur compounds shown inhibition of Gram-positive *S. aureus*, none of the studied extracts demonstrated antibacterial activity against Gram-negative *E. coli*. Thus, based on the extraction technique, solvent types, concentration, and the extracted bioactive components in the extracts, the authors who examined the antibacterial activity of *A. ursinum* extracts observed varying antimicrobial activity [14,43,52]. The bacterial strain affects the antibacterial action as well. According to Sapunjieva et al. [53], a 70% ethanol extract of *A. ursinum* has more potent antibacterial properties against Gram-positive bacteria (*L. monocytogenes* and *S. aureus*) than Gram-negative bacteria (*E. coli* and *Salmonella enterica*). Other researchers looked at the antibacterial activity of *A. ursinum* in water, methanol, and ethanol–water extracts against *S. aureus*, *E. coli*, *B. subtilis*, *P. mirabilis*, and *S. enteritidis* at varying concentrations. No meaningful comparison can be established because the methanol extract of *A. ursinum* showed no antibacterial potential, whilst the water extract only showed antimicrobial action against *B. subtilis* [14,54]. In other studies, it was found that the utilization of chloroform extract produced the most effective antibacterial activity against Gram-positive bacteria, while the antimicrobial activity of water and methanol extracts did not differ significantly [55]. Appropriate extraction methods that maintain the antibacterial properties must be performed to separate the molecules of interest for different usages.

### 2.5. Antitumor Cytotoxic Activity of A. ursinum Extracts

The in vitro cytotoxic effect of *A. ursinum* extracts was assessed against two breast cancer cell lines (T47D and MDA-MB-231) and two colorectal cancer cell lines (DLD-1 and HT29) 48 h after administration, using the MTT assay. Both breast and colorectal cancers are known to respond to diet-related factors, suggesting that foods could aid in their prevention [56,57]. Additionally, plant-derived compounds might become valuable complementary therapies for both BC and CRC [58,59]. However, the high heterogeneity of tumors—across cancer types and even within the same type—contributes to varying sensitivities to natural compounds. To our knowledge, this is the first time wild garlic extracts were tested on breast cancer cell lines and DLD-1 and HT29 cells.

**Figure 3:**
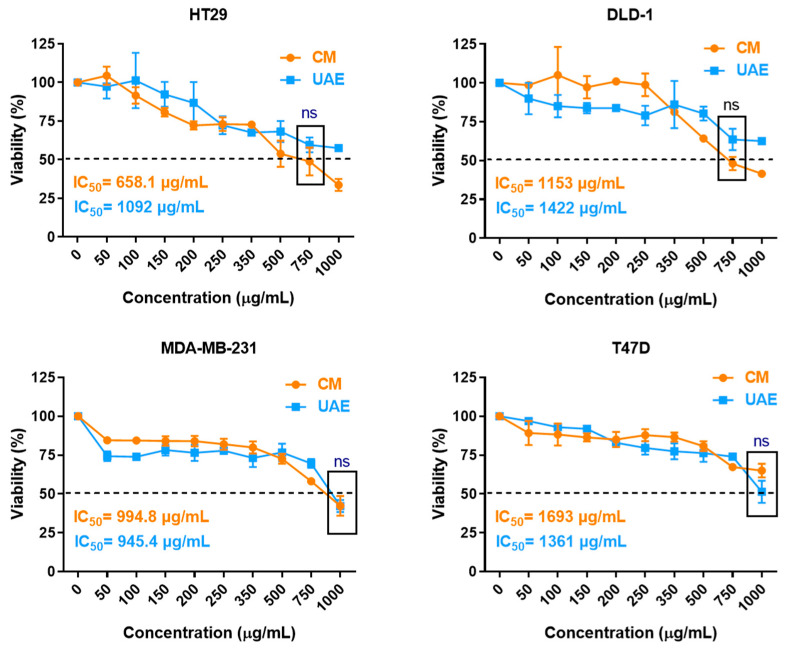
Cytotoxic activity of *Allium ursinum* leaves extract against HT29, DLD-1, MDA-MB-231, and T47D cell lines at 48 h after administration: viability curves and IC_50_ values for each extract on each cancer cell line. Data are represented as means ± standard deviation (SD) from three experiments. Statistical significance of differences between the two extracts for each cell line was evaluated using the Mann–Whitney U test (ns, *p* > 0.05).

Even though a dose-dependent decrease in cell viability was observed for both CM and UAE extracts on all four cell lines (Figure 3), the cytotoxicity of the extracts is rather moderate when analyzed in terms of general pharmacologic standards. The lowest IC_50_ values of 658.1 µg/mL and 755.8 µg/mL were obtained for the CM extract against HT29 and DLD-1 cells, respectively (Figure 3). All other IC_50_ values were around 1 mg/mL or higher (Figure 3). Even though kaempferol derivates and alliin, the major constituents found in the *A. ursinum* extracts, do possess antitumor activity and thus explain these effects, their cytotoxicity is known to be lower than that of other plant-derived related compounds, such as quercetin [60] or allicin [61]. While the CM extracts seem to yield better results against colorectal cancer cells, the UAE extract appears to have stronger cytotoxic activity against breast cancer cells. However, none of these differences are statistically significant (Figure 3), indicating that adding an extra ultrasonication step to the CM procedure provides no overall benefit when the tested concentrations are equalized after extraction.

Very limited data are available in the literature on the antitumor cytotoxic effects of wild garlic extracts. Similar results of low to moderate antitumor cytotoxicity of methanolic extracts obtained from *A. ursinum* leaves were previously reported against murine melanoma B16 and sarcoma XC cancer cells [62]. Cytotoxic effects were also demonstrated against HeLa human cervical adenocarcinoma and LS174 human colon adenocarcinoma cell lines [62], AGS [63], and MKN74 gastric cells [64]. However, the different units that are used to express the concentrations of the extracts, such as percentages or molar concentrations, do not allow for a quantitative comparison between all these results.

## 3. Materials and Methods

### 3.1. Plant Material and Preparation of Extracts

Leaves from *Allium ursinum* were collected in April 2021 from the spontaneous flora in Baia Mare, Romania. The leaves were air dried at room temperature, in the dark, for four weeks, and further milled into powder with a mortar and pestle. The powdered leaves of wild garlic were spilt into 2 experimental tubes (4 g each) and left in 70% methanol (*v*/*v*) at a 12.5:1 mL/g liquid-to-solid ratio, for 24 h, in the dark, as an initial step for both extraction procedures employed in this study: conventional maceration (CM) and ultrasound-assisted extraction (UAE). For the UAE extract, the sample was further sonicated at a fixed ultrasonic power of 750 W at 40 °C in three successive 10 min cycles (Sonics Vibra-cell VC750; Sonics and Materials Inc., Newtown, CT, USA). For both extracts, an additional maceration time of 24 h, at room temperature, in the dark was applied. The obtained homogenates were centrifuged at 450× *g* for 5 min. The supernatant was collected and further filtered through a 0.45 µm Millipore (Burlington, MA, USA). The methanol was removed using a rotary evaporator at 40 °C (Heidolph Laborota 4000 Efficient; Heidolph Instruments GmbH and CO, Schwabach, Germany). The dried residues for both extracts were weighed and further used to prepare the stock solutions at a concentration of 100 mg/mL. Methanol 70% was selected as the solvent for the biochemical analysis and dimethyl sulfoxide (DMSO) for the cytotoxicity experiments, and extracts in both solvents were used in the antibacterial activity screening. Before analytical procedures were carried out, extracts were additionally filtered through a filter with a pore size of 0.22 µm. The extraction yield was calculated based on the following formula:

$$\;\;Extraction\;yield\left(\%\right)=\frac{Weight\;of\;the\;dry\;extract\;\left(g\right)}{weight\;ofthe\;sample\;used\;for\;the\;extraction\;\left(g\right)}\times 100$$

### 3.2. The Determination of Total Polyphenolic Content by the Folin–Ciocalteu Method

The total phenolic content (TPC) was spectrophotometrically determined using the Folin–Ciocalteu method, using a modified procedure from the one described by Singleton et al. in 1999 [65]. Shortly after extraction, samples (10 µL/extract) were mixed with the Folin–Ciocalteu reagent (120 µL) and aqueous Na_2_CO_3_ (7.5% in water) (340 µL). The final mixture was allowed to stand for 60 min in the dark, then at room temperature, and then TPC was determined via colorimetry at 750 nm. The results were expressed as mg of gallic acid equivalent (GAE) per 100 mL of extract, based on a standard curve for GA. Each sample was prepared in triplicate.

### 3.3. The Determination of Total Flavonoid Content by the AlCl_3_ Method

The total flavonoid content (TFC) of the methanol extracts obtained using UAE and CM was determined using a spectrophotometric method based on the formation of a flavonoid–aluminum complex, as described by Zhishen [66]. Briefly, each extract was separately mixed with 5% NaNO_2_ (90 µL), AlCl_3_ (90 µL), NaOH (600 µL), and distilled water (720 µL) and the absorbance of each sample was measured at 510 nm. The results were expressed as mg of quercetin equivalent (QE) per 100 mL of extract. Each experiment was performed in triplicate.

### 3.4. The Identification and Quantification of Polyphenolic and Organosulphur Compounds via LC/ESI+-MS

The identification of phenolic and organosulphur compounds was performed via liquid chromatography (LC) analysis using an Agilent 1200 system (Chelmsford, MA, USA) that was equipped with a binary pump delivery system LC-20 AT (Prominence), a degasser DGU-20 A3 (Prominence), and a diode array SPD-M20 UV–VIS detector (DAD) (Shimadzu Scientific Instruments, Columbia, MD, USA). Separation of the compounds was achieved in an Eclipse XDB C18 column (4 μm, 4.6 × 150 mm). The mobile phases consisted of solvent A which contained bidistilled water and 0.1% acetic acid/acetonitrile (99/1) *v*/*v*, while solvent B contained acetonitrile and 0.1% acetic acid. The gradient elution system was programmed as following: 0–2 min, isocratic with 5% (*v*/*v*) eluent B; 2–18 min, linear gradient from 5% to 40% (*v*/*v*) eluent B; 18–20 min, linear gradient from 40% to 90% (*v*/*v*) eluent B; 20–24 min, isocratic on 90% (*v*/*v*) eluent B; 24–25 min, linear gradient from 90% to 5% (*v*/*v*) eluent B; and 25–30 min, isocratic on 5% (*v*/*v*) eluent B. The flow rate was set to 0.5 mL/min and column temperature was maintained at 25 °C. The chromatograms were monitored at 340 nm. Identification of the compounds and peak assignments were performed, using their retention time, UV–VIS analysis, and mass spectra, compared against commercial standards (chlorogenic acid, caffeic acid, quercetin-rutinoside, quercetin-glucoside, ellagic acid, and myricetin), and previously published literature. For the mass spectrometric measurements, a single quadrupole 6110 mass spectrometer (Agilent Technologies, Santa Clara, CA, USA) equipped with an ESI probe was used. Measurements were performed in the positive mode with an ion spray voltage of 3000 V and a capillary temperature of 350 °C. Data were collected in the full scan mode within the range from 280 to 1000 *m*/*z*. For the quantification of the compounds, 2 standard curves were used: gallic acid (concentrations between 10 and 100 µg/mL) for alliin and rutin (concentrations between 10 and 100 µg/mL) for flavonols.

### 3.5. Evaluation of the Antioxidant Activity by CUPRAC and Hydrogen Peroxide Scavenging Assays

Two antioxidant activity assays, the cupric reducing antioxidant capacity (CUPRAC) and hydrogen peroxide scavenging assays, were used in this investigation. The cupric Ion reducing antioxidant capacity assay (CUPRAC) is based on the reduction in the copper–neocuproin complex by an antioxidant. Briefly, 1 mL of 1.0 × 10^−2^ M CuCl_2_·H_2_O and 1 mL of 7.5 × 10^−3^ M neocuproine in urea buffer at pH 7 were mixed with (1.0 − x) mL of pH 8 standard buffer and (x) mL extract or standard antioxidant solution. The mixture was left to stand at room temperature for exactly 30 min. The absorbance was recorded using a spectrophotometer (JASCO V-630 series, International Co., Ltd., Tokyo, Japan) at 450 nm against the blank. A standard curve was prepared using different concentrations of Trolox and the results were expressed as µmol Trolox/mL.

The hydrogen peroxide scavenging assay is based on the ability of the extracts to scavenge hydrogen peroxide (H_2_O_2_) [67]. Briefly, a solution of hydrogen peroxide (40 mM) was prepared in phosphate buffer (0.1 M pH 7.4). Extracts (0.6 mL) in distilled water were added to phosphate buffer solution (1 mL). After a 10 min incubation, the absorbance was determined at 230 nm, against a blank solution (phosphate buffer without H_2_O_2_). The results were expressed as µmol Trolox equivalents per liter sample (TE µmol/L). The percentage of hydrogen peroxide scavenging is calculated as follows:

% scavenged (H_2_O_2_) = [(Ai − At)/Ai] × 100

where Ai is the absorbance of control, and At is the absorbance of test.

### 3.6. Antibacterial Activity Screening by the Agar-Well Diffusion Method

For the bioassay, five pathogenic bacterial strains were used, among which three were Gram-positive (*Staphylococcus aureus* ATCC 25923, *Enterococcus faecalis* ATCC 29212, and *Streptococcus mutans* ATCC 25175) and two were Gram-negative (*Porphyromonas gingivalis* ATCC 33277 and *Escherichia coli* ATCC 25922) (ATCC—American Type Culture Collection, Manassas, VA, USA). All bacterial strains were cultured in nutrient agar media in Petri dishes, according to the manufacturer’s instructions.

In vitro antibacterial screening of the plant extracts was carried out using the disk-diffusion method. After being cultured for 24 h on nutrient agar medium, each bacterial strain was diluted to 0.5 McFarland in sterile physiological serum. Sterile swabs soaked in these microbial suspensions were used to inoculate each Petri dish by spreading it over the entire surface of the solid culture medium (Müeller Hinton-Oxoid Agar). The Petri dishes were dried for 20 min at 37 °C and 5 mm diameter wells were carved in the agar using cut sterile pipette tips. The wells were then filled with sterile cotton beads. Each bead was loaded with 100 µL of each extract (note 1—CM extract in methanol 70%; 2—CM extract in DMSO; 3—UAE extract in methanol 70%; 4—UAE extract in DMSO) and two controls labeled M (methanol 70%) and D (DMSO). Gentamicin 10 mcg (CN10) disks were used as positive controls against all five bacterial strains. Incubation was carried out for 24 h at 37 °C (Salvis Incuccenter incubator IC400, SalvisLab, Rotkreuz, Switzerland). A ruler was used to measure the diameter of the inhibition zone (DIZ), where a larger diameter indicated greater bacterial sensitivity with respect to antibacterial substances [42,68]. The potential of inhibition (PI) of each extract on each bacterial strain was classified into four categories: strong inhibition (+ +, DIZ < 15 mm), medium inhibition (+ ±, DIZ = 10–15 mm), slight inhibition (+ −, DIZ < 10 mm), and no inhibition (− −, DIZ = 0 mm). All experiments were performed in biological triplicates.

### 3.7. Evaluation of the Antitumor Activity Using the MTT Cytotoxicity Assay

Two human breast cancer cell lines (T47D and MDA-MB-231) and two colorectal carcinoma cell lines (DLD-1 and HT29) were used in this study. All cell lines were cultured in RPMI-1640 media, supplemented with 10% PBS, 1% penicillin–streptomycin and 1% glutamine. Additionally, the T47D media was supplemented with 0.2% insulin. Cell lines were maintained in a humidified atmosphere containing 5% CO_2_, at 37 °C, in a cell culture incubator. The stock solutions of wild garlic extracts (100 mg/mL) were prepared in DMSO as the solvent and subsequently diluted with the culture medium. All cell lines were obtained from the European Collection of Authenticated Cell Cultures (ECACC) and the cell culture reagents were purchased from Sigma-Aldrich, Saint Louis, MO, USA.

The cytotoxic effects of both wild garlic extracts on all four cell lines were assessed using the 3-(4,5-dimethylthiazol-2-yl)-2,5-diphenyltetrazolium bromide (MTT) assay according to the manufacturer’s protocol. Briefly, cells were seeded in 96-well plates at an initial density of 2 × 10^4^ cells/well. After 24 h, the media was replaced with the two wild garlic extracts in nine successive concentrations (50, 100, 150, 200, 250, 350, 500, 750, and 1000 µg/mL) in fresh media, each in six technical replicates. After a 48 h incubation period at 37 °C in 5% CO_2_, the supernatant was removed and 100 mL of the MTT solution (1 mg/mL) was added. After 1 h, the MTT solution was removed, 150 µL DMSO was added to each well to solubilize the formed formazan and the absorbance was measured spectrophotometrically at a wavelength of 570 nm, using a multi-mode reader (Synergy HTX, BioTek, Winooski, VT, USA).

Based on the absorbance values, viability was calculated as the fraction of viable cells compared with the untreated control. The half maximal inhibitory concentration (IC_50_) values on each cell line was calculated in GraphPad Prism Software Version 7 (GraphPad Software, Inc., Avenida de la Playa, La Jolla, San Diego, CA, USA), using the log(inh) vs. normalized response nonlinear regression equation. The statistical significance of differences in antitumor potential between the two extracts for each cell line was evaluated using the Mann–Whitney U test, based on viability at the concentration where at least one extract achieved 50% inhibition. A *p*-value of <0.05 was considered statistically significant.

## 4. Conclusions

*Allium ursinum* extracts exhibit significant antioxidant and antibacterial properties, supporting their potential as complementary treatments for microbial infections and conditions associated with oxidative stress. However, the extracts exhibit only low to moderate antitumor activity in vitro, likely due to the absence of highly cytotoxic compounds. These findings highlight *A. ursinum* as a promising source of natural bioactive compounds for medical applications, warranting further exploration.

As anticipated, adding an ultrasonication step in the conventional maceration procedure increases the extraction yield of bioactive constituents, which, in turn, enhances both the antioxidant and antibacterial activities of the extracts. Therefore, by carefully selecting extraction parameters like ultrasonic power and frequency, it is possible to improve extraction efficiency without degrading polyphenols. However, when concentrations are equalized after extraction, no additional benefits from ultrasonication are observed in terms of efficacy during use.

## Figures and Tables

**Table 1:** Tentative identification and quantification of polyphenols and sulfoxides in the extracts of *A. ursinum* leaves obtained through conventional maceration (CM) and ultrasound-assisted extraction (UAE).

Peak No.	Rt (min)	UV λmax (nm)	[M + H]+ (*m*/*z*)	Compound	Subclass	CM * (mg/mL)	UAE+CM * (mg/mL)
1	2.91	230	178	Alliin	sulfoxide	4.641	5.575
2	11.38	260, 350	757, 611, 449, 287	Kaempferol-glucosyl-glucosyl-rhamnoside	flavonol	5.068	5.967
3	12.65	260, 351	611, 449, 287	Kaempferol-diglucoside	flavonol	1.759	2.259
4	14.65	261, 350	595, 287	Kaempferol-galactosyl-rhamnoside	flavonol	0.925	1.120
5	15.56	261, 350	595, 287	Kaempferol-rutinoside	flavonol	3.050	3.450
6	16.05	250, 350	611, 303	Quercetin-rutinoside	flavonol	1.248	1.572
7	17.15	260, 351	449, 287	Kaempferol-glucoside	flavonol	0.489	0.620
			Total	polyphenols		12.539	14.988
			Total	compounds		17.181	20.563

Rt—retention time; UV λmax—wavelengths of maximum absorption; [M + H]+—molecular ion; *—quantification results are expressed as milligram of fraction equivalent (gallic acid—thiosulphinate and rutin—flavonols) per mL sample.

**Table 2:** Antimicrobial activity of *Allium ursinum* leaves extracts obtained through conventional maceration (CM) and ultrasound-assisted extraction (UAE) on Gram+ and Gram− pathogenic microorganisms.

Antibacterial Activity	Extracts								Controls					
	MET CM (1)		DMSO CM (2)		MET UAE (3)		DMSO UAE (4)		MET (M)		DMSO (D)		Gentamicin	
	DIZ (mm)	PI	DIZ (mm)	PI	DIZ (mm)	PI	DIZ (mm)	PI	DIZ (mm)	PI	DIZ (mm)	PI	DIZ (mm)	PI
**Gram-positive**														
*Staphylococcus aureus*	10.0	+ ±	10.0	+ ±	11.9	+ ±	12.0	+ ±	0	− −	0	− −	20	++
*Enterococcus faecalis*	8.5	+ −	11.0	+ ±	8.5	+ −	12.1	+ ±	0	− −	0	− −	17.8	++
*Streptococcus mutans*	11.9	+ ±	12.0	+ ±	11.5	+ ±	12.5	+ ±	0	− −	0	− −	18.2	++
**Gram-negative**														
*Porphyromonas gingivalis*	12.9	+ ±	12.0	+ ±	15.1	++	10.5	+ ±	0	− −	0	− −	14.5	+ ±
*Escherichia coli*	8.0	+ −	17.1	++	8.5	+ −	15.0	++	0	− −	0	− −	18.5	++

MET—methanol 70% (solvent); DMSO—dimethyl sulfoxide 100% (solvent); DIZ—diameter of inhibition zone; PI—potential of inhibition.

## Data Availability

Data is contained within the article.
